# Patients’ suggestions for improvements to text-based e-consultations. An online survey of users of the national health portal in Norway

**DOI:** 10.3389/fdgth.2024.1459684

**Published:** 2024-12-09

**Authors:** Eli Kristiansen, Helen Atherton, Trine Strand Bergmo, Paolo Zanaboni

**Affiliations:** ^1^ Norwegian Centre for E-health Research, University Hospital of North Norway, Tromsø, Norway; ^2^ Department of Clinical Medicine, UiT The Arctic University of Norway, Tromsø, Norway; ^3^ School of Primary Care, Population Health and Medical Education, University of Southampton, Southampton, United Kingdom

**Keywords:** remote consultation, e-consultation, primary healthcare, general practitioner, patient feedback

## Abstract

**Background:**

In recent years, text-based e-consultations have been widely implemented in general practice and are appreciated by patients for their convenience and efficiency. Policymakers aim to enhance patient access to clinical services with the general practitioner (GP) through text-based e-consultations. However, concerns are raised about their efficiency and security. We aimed to investigate users’ perceptions of potential improvements in the text-based e-consultation service provided by the national health portal in Norway.

**Method:**

We conducted an online survey among users of text-based e-consultations with the GP on the national health portal Helsenorge. The survey was available from January-February 2023 and consisted of 20 questions. This study focused on the free-text answers to the question “*Do you have any suggestions to improve the service?”* The framework method was used for a thematic analysis of the answers.

**Results:**

The analysis of 2,954 free-text answers from users of the national e-consultation service resulted in six areas where suggestions for improvement were expressed. According to users, the service would benefit from: (1) a better set-up to facilitate the formulation of the patient's problem, (2) better value for money (in regards to both price and quality), (3) faster response time, (4) improved information and predictability about the status of the e-consultation (e.g., if it is received and when to expect an answer), (5) improvement in technical issues, and (6) improvement of access to dialogue-based services to replace or complement e-consultations.

**Conclusion:**

The analysis of users’ suggestions for improvements to the e-consultation service emphasised the need to customise the service to address individual patient needs. Users found a one-size-fits-all approach with mandatory questions, fixed pricing, and inflexible response times less appreciated. Some also felt forced to rely on e-consultations due to the perceived poor availability of other GP services. This highlights the importance of perceiving e-consultations not as a replacement for dialogue-enabled services, but rather as a potentially efficient addition, ensuring a well-tailored setup for appropriate patient use.

## 1 Introduction

Digital health services in primary care have, in recent years, been increasingly implemented worldwide to improve patient access to care (1). The COVID-19 pandemic had a major impact on their rapid implementation (2). One of these digital services is text-based e-consultation, an asynchronous service used by patients to send a written clinical inquiry to their GP (3). Following the large-scale adoption, the use of text-based e-consultations has remained relatively high in post-COVID-19 times (4).

Studies show that text-based e-consultations are appreciated by users (3, 5, 6) for being more convenient, increasing the availability of clinical GP services for patients and saving time for both patients and GPs compared to office appointments (7–10). The service is also perceived as easy to use (8), and patients express satisfaction with the quality of care provided through text-based consultations (11). However, studies also suggest that text-based e-consultations with the GP might increase patients’ demand for GP services and the workload for GPs (8, 12, 13), making it less efficient. Text-based e-consultations’ user surface is constantly developing as more knowledge of the use and functionality of the systems is produced. Several studies have been conducted on patients’ views and use of e-consultations and similar text-based triage online services (12, 14–16). Still, there is a need for studies that investigate whether the service gives patients effective, efficient, and satisfactory clinical help in a normalised post-COVID-19 setting when social distancing is not the main reason for use and the service is broadly implemented across the population.

In Norway, general practice is organized so that every resident is assigned a regular GP. Lately, GPs are experiencing an increased workload (17). Policymakers’ aim with digital healthcare services (including text-based e-consultations) is to give patients better access to clinical GP services (18). Offering e-consultations is voluntary for the GPs. A specific tariff for e-consultations was introduced in 2016, and GPs receive the same reimbursement as for office visits. Patients pay the same out-of-pocket fee for e-consultations as for office visits. GPs can charge the patient more if they answer the e-consultation in the evening. The e-consultation must include a medical assessment of the patient's request, and the GP is expected to answer within 5 working days (19).

Helsenorge is the national online health portal in Norway, where residents can find information about healthcare services and access several digital services after a secure login. GPs can choose to offer up to four digital services for citizens: (1) an electronic booking service to make appointments with the GP; (2) an electronic prescription service to request renewal of prescriptions; (3) text-based e-consultations for clinical enquiries, and (4) a service for text-based non-clinical enquiries to the GP office (20). Using the text-based e-consultation service in Helsenorge is free for the GP.

At the beginning of 2022, around 70% of all GPs used one or more digital services in Helsenorge, and approximately 50% offered and used the text-based e-consultation service (21). There are no official numbers on patients’ use on text-based e-consultations in Helsenorge. However, official data shows that, at the time of the study (beginning of 2022), e-consultations (including text-based, video and telephone consultations) represented 20%–30% of all consultations done with the GP (4).

The objective of this study was to explore patients’ suggestions for potential improvement in the text-based e-consultation service in Norway. The study was performed on the national health portal Helsenorge, which hosts the majority of text-based e-consultations conducted between patients and GPs nationwide. To investigate the service's potential improvements, we asked e-consultation users to provide feedback on what could be done to improve the service.

## 2 Methods

### 2.1 Data collection

We conducted a retrospective cross-sectional study through an online survey of users of text-based e-consultations with the GP. The survey was developed by the study authors, who have expertise in digital health and primary health care. All patients who sent a text-based e-consultation to their GP through the online national health portal Helsenorge from Monday 30/01/2023 through Sunday 19/02/23 received an invitation through a pop-up window reading: “*Do you want to participate in research about text-based e-consultations*?”. The respondents were informed that participation in the survey was voluntary and anonymous. Consent was given by clicking ‘I agree to be a part of the survey’ in the pop-up window. Respondents were then redirected to an external webpage where the survey was presented. Altogether, the survey consisted of 20 questions including the non-mandatory free-text question: “*Do you have any suggestions to improve the service?”.* The current study is based on the free-text answers provided to that last question. The online data collection solution Questback Essential (Oslo, Norway) was used to collect answers to the survey.

**Figure 1:**
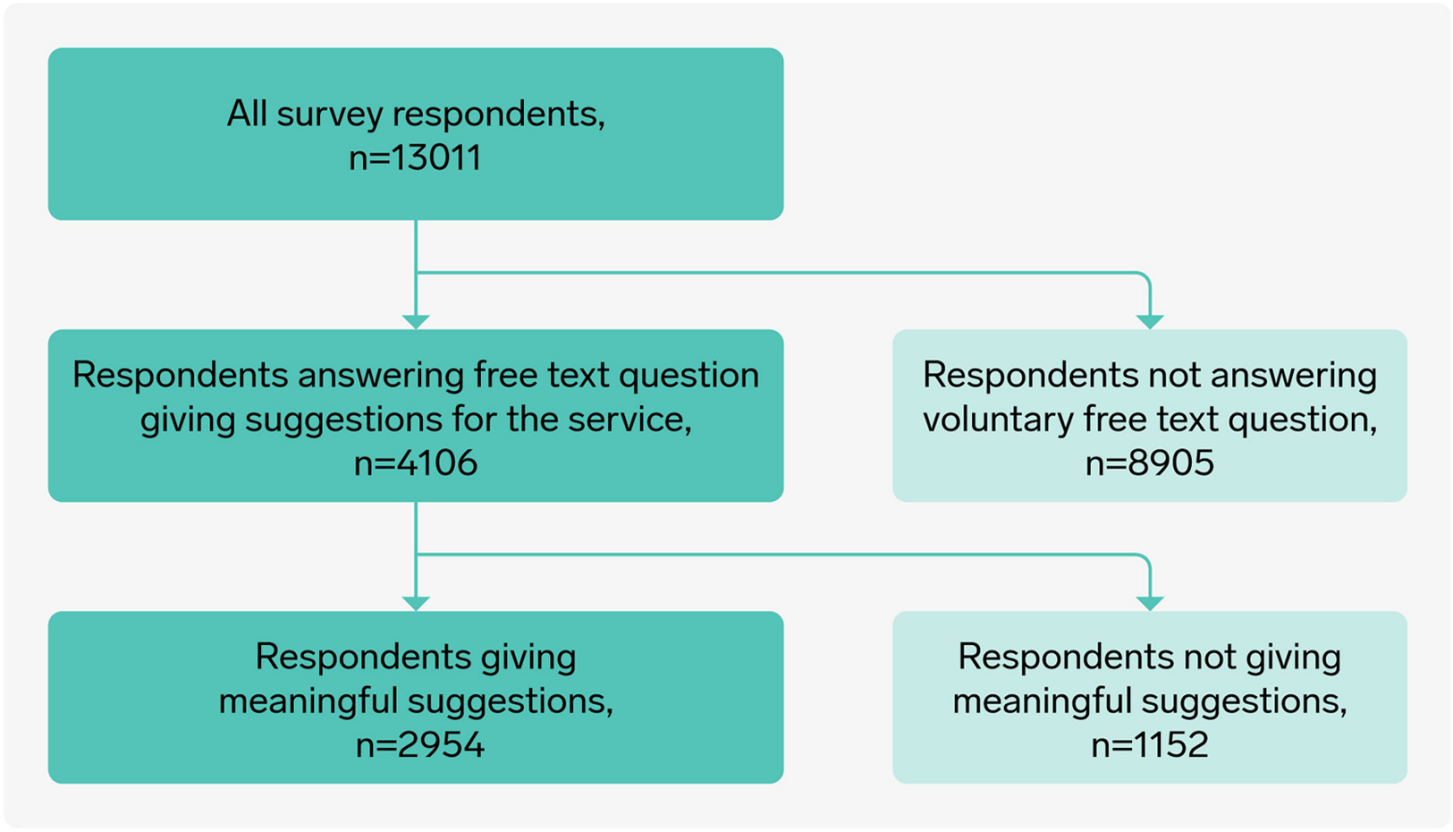
Flow chart of respondents to free text answer.

A total of 13,658 respondents answered the survey. The answers from 647 respondents were removed due to a technical mistake, leaving with a sample of 13,011 respondents. Of these, 4,106 wrote a free-text answer to the question about potential for improvements. After removing all answers without meaningful suggestions for service improvement (e.g., no, don't know, I am very satisfied with the service) the final data set that was analysed included 2,954 answers (Figure 1).

### 2.2 Data analysis

We calculated summary descriptive statistics of the respondents for the whole study and the sample that answered the free-text question. Chi-square test was used to examine if there were indications of significant differences between those that answered the free-text question and the remaining respondents of the survey. The analysis was done using SPSS version 25.

A thematic analysis was conducted through the framework method (22, 23). The framework method is designed to suit a multidisciplinary team and is appropriate for analysing free-text answers that cover a specific issue (23). The free-text answers were taken from a quantitative survey, which imposed some limitations on the data set, as we could not ask follow-up questions or further explore interesting topics that emerged. However, the free-text answers were rich in content, and a qualitative analysis was considered of high value. The analysis team had different backgrounds, including technology, health, social science and economics, but their primary common academic interest was digital health. The first author (EK) read through all 4,106 answers and coded them into 22 categories in line with the framework method. After the initial coding, EK read through all the categories to ensure that the categories and placements of answers were correct. Then, the categories and answers were modified, merged, and divided into themes. PZ and TSB helped with the categorisation and identification of final themes. HA read through and modified discussions and results sections. The final analytical framework consisted of 19 categories grouped into 6 themes representing areas for potential improvement. Citations in results are free text answers from the survey, translated to English. Excel for Microsoft 365 MSO (Version 409) was used for analysing the data.

### 2.3 Study setting

At the time of the survey (January 2022), GPs could choose between two different options for set-up of text-based e-consultations on the national health portal Helsenorge. The first option comprised of five mandatory questions: (1) What can the GP help you with? (max 50 characters), (2) Describe the symptoms or ailments you have (max 350 characters), (3) Have you done any measurements yourself? Yes/No – If yes, what was the result of the measurements? (max 50 characters), (4) How do the health challenges affect your everyday life? (max 200 characters), (5) Have you tried to treat your problem yourself? Yes/No—If yes, which treatment have you tried, and what effect did it have? (max 100 characters). Alternatively, the second option consisted of one text box (max. 1,000 characters) where the patient was asked to write a health-related enquiry to the GP (19, 20). Uploading up to three attachments (allowed formats, such as JPG, PNG, or PDF) was possible. Since Helsenorge did not offer a service to collect patient payments, GP offices had to use payment systems from private providers to collect patient payment. This was typically done either via SMS with a link to the payment system or by sending an invoice.

### 2.4 Ethical approvements

The study and the procedure for handling the data were approved by the Data Protection Officer of the University Hospital of North Norway (#03057). According to the Norwegian Act on Medical and Health Research §2 and §4, the study did not require approval from the ethics committee.

## 3 Results

Of the 13,011 respondents who answered the whole survey, 2,954 respondents answered the free text question with a meaningful answer. All patient characteristics were significantly different between the group of respondents who answered the free-text question and the ones who did not (*p* < 0.001) (Table 1). A larger proportion of respondents who answered the free-text answer were dissatisfied or very dissatisfied with the service compared to the ones that did not answer (8% vs. 0.9%). The respondents who answered the free text question also had higher education level than the remaining respondents. Finally, 18.1% of the respondents answering the free-text had more than 10 e-consultations in the last 12 months compared to 12.3% of the ones that did not answer the free-text question.

**Table 1:** Characteristics of respondents, *N* = 13,011.

	Respondents whose answers are included in free-text analysis		Respondents who did not answering free text answers		All respondents	
	*n* = 2,954		*n* = 10,057		*N* = 13,011	
	*n*	%	*n*	%	*N*	%
Gender						
Female	2,083	70.5	6,976	69.4	9,059	69.6
Male	837	28.3	3,011	29.9	3,848	29.6
Other	34	1.2	70	0.7	104	0.8
Age						
16–25 year	203	6.9	620	6.2	823	6.3
26–40 year	905	30.6	2,690	26.7	3,595	27.6
41–55 year	1,130	38.3	3,634	36.1	4,764	36.6
56–70 year	579	19.6	2,544	25.3	3,123	24.0
over 71 years	137	4.6	569	5.7	706	5.4
Highest completed education level						
10-years primary school or less	162	5.5	633	6.3	795	6.1
Upper secondary school	416	14.1	2,337	23.2	2,753	21.2
Vocational school	553	18.7	1,617	16.1	2,170	16.6
University less than 4 years	732	24.8	2,424	24.1	3,156	24.3
University more than 4 years	1,026	34.7	2,849	28.3	3,875	29.8
Other	65	2.2	197	2.0	262	2.0
Travel time to GP office						
0–30 min	2,227	75.4	7,928	78.8	10,155	78
30–60 min	538	18.2	1,640	16.3	2,178	16.7
1–2 h	124	4.2	344	3.4	468	3.7
More than 2 h	65	2.2	145	1.4	210	1.6
Appointments at the GP office last 12 months						
0–3 appointments	1,282	43.4	4,954	49.3	6,236	47.9
4–9 appointments	1,336	45.2	4,236	42.1	5,572	42.8
10–19 appointments	273	9.2	756	7.5	1,029	7.9
20 or more	63	2.1	111	1.1	174	1.4
E-consultations last 12 months						
1–3 e-consultations	1,334	45.2	5,312	52.8	6,646	51.1
4–9 e-consultations	1,084	36.7	3,523	35.0	4,607	35.4
10–19 e-consultations	385	13.0	973	9.7	1,358	10.4
20 or more	151	5.1	249	2.5	400	3.1
First e-consultation ever						
Yes	319	10.8	1,439	14.3	1,758	13.5
All in all, how satisfied were you with contacting the GP through an e-consultation today?						
Very satisfied	992	33.6	5,503	54.7	6,495	49.9
Satisfied	1,095	37.1	3,323	33.0	4,418	34.0
Neither satisfied or not	631	21.4	1,145	11.4	1,776	13.6
Dissatisfied	172	5.8	49	0.5	221	1.7
Very dissatisfied	64	2.2	37	0.4	101	0.8

The 2,954 free-text answers differed both in length and content. Some respondents only wrote a few words (e.g., *faster response*), while others wrote longer texts. The mean number of characters in the answers was 150 (min:5, max:3,922).

**Figure 2:**
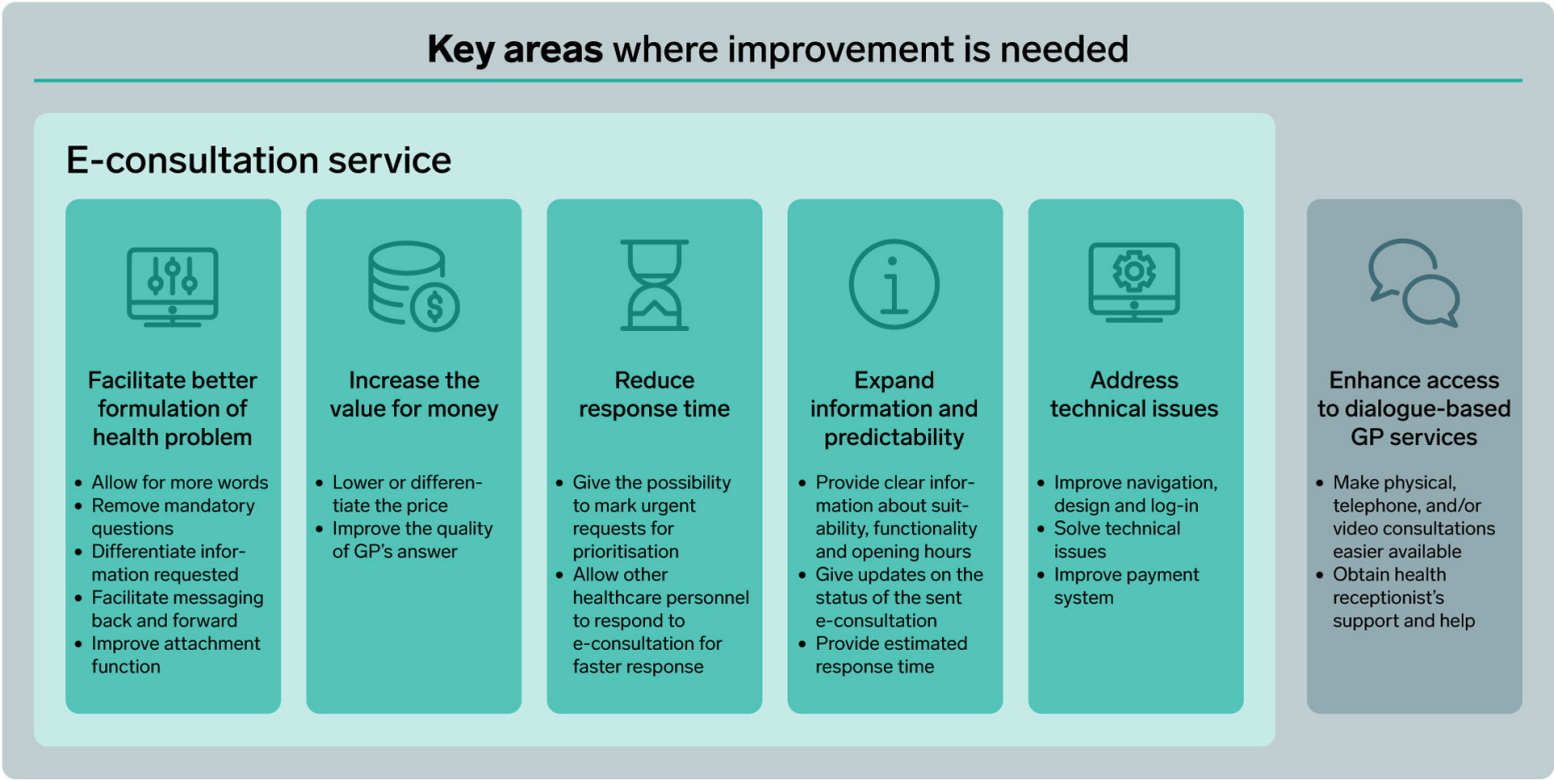
Suggestions for potential improvement within each key area.

The analysis revealed six areas where patients expressed potential for improvements in the e-consultation service. Figure 2 lists the specific suggestions for improvement that could be made within each area.

### 3.1 Facilitate better formulation of the health problem

The respondents suggested improvements in how the e-consultation service is set up, allowing patients to formulate their health problems more clearly to the GP. The strict limitations on characters/words made it hard for the patients to describe their problems adequately. Many users did not appreciate using the structured form with five questions as they did not find it suitable or necessary for their request. Others pointed out that the questions were somatic-oriented and not suitable for mental issues.

The introductory questions that have come up lately are completely meaningless. The GP knows my health challenges, and I don't need to describe them as if it were the first time we talked about them (male 26–40 years old).

The respondents emphasised that e-consultations should only request information perceived as essential for their health problem and differentiation regarding what information was requested from the patient based on the patient's need for help was suggested. Many felt that they had to repeat information already shared with the GP in earlier appointments. A common suggestion was to create different setups for different enquiries, such as for sick certificates and other forms of certification, for managing long-term chronic conditions, or for straightforward yes-no questions directed to the GP (e.g., brief clarification right after a doctor's appointment).

[I don’t like that] The questions are the same regardless of the enquiry’s concerns. If e-consultations are to be a real option, an assessment of what you need from the e-consultation should come as the first question (female 41–55 years old).

The respondents also suggested different solutions for a more dynamic communication with the GP. Options that were mentioned were synchronous chats or the possibility to reply directly to the answer provided by the GP. This would mean removing the GP's possibility to close the e-consultation request. Finally, better functionality for attachments, including the possibility of attaching a video, was suggested to help the patients describe their problems adequately.

### 3.2 Increase the value for money

The free-text answers often focused on the patient's assessment of the value of the service. This was explicitly related to the quality of the clinical help they received, the price of the service, and how long they had to wait for the answer. Several users described that they had experienced poor quality in the clinical help provided through e-consultations. They felt that the responses were rushed and lacked thorough consideration from the GP, often addressing only isolated aspects of the patient's condition, thus diminishing the perceived value of the service.

Answers that are short and superficial due to little time from the doctor can lead to uncertainty and thus poorer treatment/effect of a change in medication, etc. If the doctor wants you to not worry about something, the patient must understand what to think about it. Perhaps a doctor’s appointment would be better for these issues, but how the doctors word themselves in an e-consultation is also very important (male 41–55 years old).

Moreover, the respondents expressed dissatisfaction with the cost of the service relative to the perceived value. They highlighted that the service should not cost as much as a face-to-face appointment, as it was not the same as a meeting with the GP. Some criticised the additional cost of e-consultations that were answered in the evening. It is solely the GP's choice to send an answer in the evening and not something the patient could decide for. Many also proposed that different prices should be implemented based on the complexity of the e-consultation (e.g., straightforward yes-no questions should incur lower costs than medical evaluations).

Strangely, it [e-consultation] costs the same as a doctor’s appointment, and you may also have to pay for an evening supplement. We are not given the choice of when they will read the consultation. An e-consultation is, of course, not as good an offer as meeting the doctor physically. Therefore, it should not cost as much either - but it could be a good offer to reduce the burden of the pressure at the doctor’s office and the problem of taking time off work to go to the doctor (female 26–40 years old).

### 3.3 Reduce response time

The respondents wanted faster responses and were unsatisfied with waiting up to five working days for an answer to their e-consultation. It was suggested that redistributing the responsibility of answering e-consultations within the general practice (to include other GPs and health receptionists) could enhance response time.

I sent the same message to the doctor’s office on Thursday and yesterday. If you need to extend your sick leave, you cannot wait and see; you must send it to the employer the same day. I think it could help to have the same thing for sick notes as we have when we need to renew a prescription so that sick notes and other questions are not getting mixed up. In addition, it has become much more challenging to talk to the doctor’s office, and you don't get an answer from Helsenorge for 4–5 days (female 56–70 years old).

Additionally, users expressed a desire to flag particularly important or urgent e-consultations for prioritisation in the GP’s inbox and possibly receive a faster response.

A sort of self-ranking or triage of the e-consultation: whether you need an answer within 12 h/24 h/48 h/etc. Many things are not urgent, and people can be willing to wait for some days, and I think many people are honest about that. While something, e.g., a prescription/new medicine or new diseases and symptoms, is more urgent (male 26–40 years old).

### 3.4 Expand predictability and information about the service

The users wanted a more predictable e-consultation service, regarding both *when* the service was available and *updates* on the status of their sent e-consultations.

Opening hours for e-consultation should either be 24/7 or fixed and specified on the webpage (female 26–40 years old).

Some users wrote about experiencing how the e-consultation service would unexpectedly close without indicating when it would reopen. Others reported occasions when the service disappeared entirely from the webpage.

Sometimes, the e-consultation service is closed because the doctor has been on a course or, for other reasons, is not at work. Other times, it has taken a long time for her to respond. I miss a function for “the doctor is absent, the next consultation is Monday” and a message saying “e-consultation received, under treatment”. Then you don't have to wonder or nag the doctor’s secretary about it (female 41–55 years old).

Predictability on the status of sent e-consultations was reported as lacking. Users recommended implementing notifications confirming that the e-consultation was received in the system and when it was read by the GP. They also recommended that an estimated response time could be provided based on the GP's current workload. This feedback resonated with the feedback from those who had not received responses to their e-consultations, leaving them uncertain whether the issue lay in the e-consultations not being successfully sent or if it was the GP that simply had not had the time to respond.

I want to get a confirmation that the doctor has read and received the e-consultation. Also, more predictability of when the e-consultation will be answered. Perhaps a status message showing “unread”, “read”, “processing your case”, or “solved” so you can get a little hint that the doctor has seen the message or not, at least if the response time is longer than 24 h (female 26–40 years old).

The respondents also requested more information about the service, primarily about the operational aspects of e-consultations: queries about the payment process (if they had to pay for the service, how to pay for it) and information on the differences between the digital services offered by the GP. Moreover, information about the clinical appropriateness of when to use the e-consultation service was requested.

The doctors’ offices must be encouraged to have their policy clearly described on the website. Here, the details of what e-consultation can and cannot be used for can be explained with examples (female 41–55 years old).

Suggestions for improvement might include having more contact options and a better explanation on Helsenorge and the GP’s websites about how to use e-consultation and what the GP prefers (male 41–55 years old).

### 3.5 Address technical issues

There were reported difficulties in navigating the health portal and finding the appropriate section for submitting e-consultations.

I didn't even find the “button. ” When I logged in, it didn't appear in the first picture. I had to go back and forth in the menu to find it (female 56–70 years old).

Other users described the need for improved webpage design, noting that the colour schemes were unsuitable for those with visual impairments. Technical glitches were also common, with users experiencing issues such as text not being automatically saved, leading to content loss when unexpectedly logged out of the site. Also, dissatisfaction with how to pay for the e-consultation was expressed, particularly regarding how payment requests were sent after e-consultations were submitted. Some users objected to additional invoice fees and preferred immediate payment for the service. Finally, there were requests for English instructions to be provided for the service.

### 3.6 Enhance access to dialogue-based GP services

Although users were asked for suggestions to improve the e-consultation service, many responses centred around alternative or complementary services to e-consultations and described dissatisfaction with the availability and accessibility of GPs and health receptionists. Some patients appeared to have used e-consultations as a substitute for traditional consultations (i.e., face-to-face consultations and telephone consultations) because these were unavailable.

I think many people see e-consultation as a safe way to talk to their doctor without necessarily having to attend an appointment and perhaps the only way to make direct contact without waiting many weeks for a doctor’s appointment. A GP can have an awful lot of patients, and it is noticeable that the workload is high and that you, as a patient, do not always get the necessary help. E-consultation at least guarantees contact with a GP, as far as I know, even if it takes several days (female 56–70 years old).

Some respondents asked for easier accessibility to physical appointments through electronic booking, while others suggested more accessible digital dialogue options, like telephone or video consultations.

I think it is problematic that e-consultations seem to completely replace telephone consultations with the doctor (male 55–70 years old).

Telephone and video were also requested as a supplement to the text-based e-consultations, suggesting that GPs could contact patients via video or telephone after receiving text-based e-consultations if the GP assessed it as necessary. Finally, some respondents expressed interest in using video and telephone consultations instead of text-based e-consultations due to the need for a real-time dialogue to address their concerns more directly.

There should be a box or section where you can tick off or add that the doctor can call the patient if the doctor sees the e-consultation and thinks it is more appropriate with a phone call or that the patient must come in for an appointment (female 26–40 years old).

E-consultations were also described as affecting the health receptionist's role, as they offered less assistance and were reluctant to assist with enquiries after the e-consultations service was implemented. The respondents explained how health receptionists encouraged patients to contact the GP through e-consultations, which was perceived as a disappointing departure from a previously valued service of real-time dialogue with easily accessible help with the health receptionist.

E-consultations have done so the health receptionist cannot and will not make any decisions. On the answering machines at the doctor’s office, they do everything they can to discourage people from calling the practice. “You can check test results, book an appointment, etc, on the website”. They also say, “You can send an e-consultation round the clock via Helsenorge”. Well, you can't do that with my doctor. She works Monday, Wednesday and sometimes Friday (female 26–40 years old).

## 4 Discussion

### 4.1 Main results and previous research

The analysis of 2,954 free-text answers from users of the national e-consultation service identified six key areas for improvement. Users suggested that the service would benefit from a better setup to facilitate formulating the patient's problem, mainly focusing on more available words. Furthermore, they wanted an increase in the value for money in terms of both price and quality. Other themes were reduced response time, improved information and predictability, technical issues being addressed, and enhanced access to dialogue-based services to complement or replace e-consultations. Many of these suggestions stemmed from users’ perceptions that the e-consultation service was too generic and not tailored to individual patient needs and expectations. Personalised care can be described as an efficient method of delivering care that minimises the burden on the patient while recognising and respecting their autonomy and expertise (24). The analysis showed that users desired a more personalised e-consultation service that catered specifically to their enquiries regarding what information was requested from them, as well as response time and cost.

Value for money can be defined as the health outcomes achieved relative to the cost borne by the patient (25). Many users did not feel like e-consultations gave good value for money. They perceived the service as too expensive and occasionally noted low quality of the response received. There is limited evidence of patients’ willingness to pay for e-consultations. In Norway, patients are charged the same fee for text-based e-consultations as they are for face-to-face consultations. This contrasts with other countries such as Canada, Denmark, and England, where patients do not incur charges for consultations, whether physical or virtual (26–28).

User dissatisfaction with fixed and mandatory questions in e-consultation forms is known (9). Our study shows how it is perceived unnecessary to provide repeated information that is already well-known to the GP. As the Norwegian GP scheme is based on continuity (29) and remote consultations are often used by frequent attenders and patients with existing health problems (30), for these patients, in particular, the structured form with mandatory questions was perceived as exhausting. Other studies show that patients using systems with free-text e-consultations are satisfied (3, 31, 32). However, health professionals perceive standardised questions as a feature that improves patient safety (7, 26). Banks (28) showed how eConsult messages in England often contained too little information from the patient, resulting in the need for a follow-up phone call to specify the information. Presumably, helping patients give the necessary information is the justification for setting up fixed questions in Helsenorge. Chatbots that help patients to communicate sufficient information in free text, thus avoiding to provide repeated information, represent a potentially valuable alternative (31).

Patients’ need for more information about the use and suitability of e-consultations is well documented (6, 9, 33, 34). Information is crucial for reducing inequity in access to digital healthcare services and minimising the risk of potentially unsafe urgent requests (33). This is especially important for those with lower levels of education, as it ensures they have a good experience while using digital tools (35). Predictability is also important for patients using e-consultations, and dissatisfaction related to the lack of status updates has been previously shown (3, 9, 32).

A fast response to text-based e-consultations might increase access to GP services and improve patient satisfaction (32). The timeframe for answers to online consultations in the UK is set by each GP practice individually, generally within 48 h (31). In Norway, it is recommended that the answer be within five working days (19). Many studies have shown that patients want a lower response time to their online consultations or eConsult (3, 9). Several respondents suggested that the patients themselves should mark their own assessment of urgency for their issue. Notably, the risk of unequal treatment with such a solution is high, and we do not consider this a sustainable solution that will provide equity.

Finally, as an input to the debate of whether e-consultations increase patients’ access to the GP, our findings indicate that some patients use the e-consultation service because other GP services (such as physical appointments and telephone) are perceived as unavailable. Difficulty in accessing other GP services, making e-consultations the only available option, has been shown in studies before (12, 28, 31). Other studies emphasise patients’ appreciation of the accessibility that digital consultations provide, and how they prefer using these over face-to-face consultations (30, 36).

### 4.2 Strengths and limitations

The study is based on a large sample of free-text answers collected through a nationwide online survey and presents a unique dataset. The survey was conducted after the COVID-19 pandemic, in a period when the e-consultation service was widely adopted and used by many Norwegian GPs, with the majority of the respondents having several experiences with using the service. A limitation of the study design is that the free-text answers did not provide opportunities to further explore interesting topics that emerged. By describing the portal and its functions, the study's findings might be more easily generalisable to other services and settings. No data is available about the Norwegian population of users of text-based e-consultations. Consequently, it was impossible to evaluate the study sample's representativeness. While most of the users who answered the survey were highly satisfied with the service (92%), the free-text respondents were less satisfied and had a higher level of education. This suggests that the sample may be somewhat biased. Despite this potential bias, the study still provides valuable insights into areas for improvement that could benefit all users. Its findings are important for understanding user satisfaction and guiding service enhancements.

### 4.3 Implications and future research

The high availability offered by text-based e-consultations is appreciated by patients in a system where the availability of GPs is perceived as a problem (5, 12). This makes the service highly demanded by the patients, and there are no indications that the demand will decrease in the future. Identifying potential areas for service improvement, as done in our study, is crucial for further development of the e-consultations service. An efficient e-consultation service should help patients understand when its use is appropriate and ensure that the necessary information is provided to the GP. This way the likelihood of needing additional in-person follow-up consultations is reduced, and patient safety enhanced (13). Quality and responsiveness of the service must be good for patients to consider the service as an equal alternative to other GP services. Since offering e-consultations is optional for the GPs, the continuity of the service will depend on how GPs perceive its efficiency. Future research should focus on identifying improvement areas from the perspective of the GPs to ensure that the service meets their needs and remains a viable option within healthcare delivery.

### 4.4 Updates on Helsenorge and the e-consultations service

Since conducting this study, the national health portal Helsenorge has improved several of the suggested functionalities described in the study, including the option to send e-consultations to a substitute GP and clearer information regarding out-of-pocket fees and response times. A solution for booking telephone appointments has also been developed, among other features. In addition, the authorities have eliminated the additional out-of-pocket fee requested to patients for e-consultations answered by GPs in the evening.

### 4.5 Conclusion

The analysis of users’ suggestions for improvements to the e-consultation system emphasised the need to customise the service to address individual patient needs. A one-size-fits-all approach with mandatory questions, fixed pricing, and inflexible response times is less appreciated by users. Additionally, a concerning finding was that users felt forced to rely on e-consultation services because the other GP services were perceived as poorly available. This highlights the importance of not interpreting e-consultations as a replacement for other dialogue-enabled services but instead considering e-consultations as a potentially efficient addition to other GP services. Enough information and a well-tailored set-up should be offered to ensure efficient use so that the patients use the service for the appropriate issues.

## Data Availability

The raw data supporting the conclusions of this article will be made available by the authors, without undue reservation.
